# Neurosurgical Considerations Regarding Decompressive Craniectomy for Intracerebral Hemorrhage after SARS-CoV-2-Vaccination in Vaccine Induced Thrombotic Thrombocytopenia—VITT

**DOI:** 10.3390/jcm10132777

**Published:** 2021-06-24

**Authors:** Florian Gessler, Ann Kristin Schmitz, Daniel Dubinski, Joshua D. Bernstock, Felix Lehmann, Sae-Yeon Won, Matthias Wittstock, Erdem Güresir, Alexis Hadjiathanasiou, Julian Zimmermann, Wolfgang Miesbach, Thomas Freiman, Hartmut Vatter, Patrick Schuss

**Affiliations:** 1 Department of Neurosurgery, University Medicine Rostock, 18055 Rostock, Germany; daniel.dubinski@med.uni-rostock.de (D.D.); sae-yeon.won@med.uni-rostock.de (S.-Y.W.); Thomas.freiman@med.uni-rostock.de (T.F.); 2 Department of Neurosurgery, University Hospital Bonn, 53127 Bonn, Germany; ann_kristin.schmitz@ukbonn.de (A.K.S.); erdem.gueresir@ukbonn.de (E.G.); alexis.hadjiathanasiou@ukbonn.de (A.H.); hartmut.vatter@ukbonn.de (H.V.); patrick.schuss@ukbonn.de (P.S.); 3 Department of Neurosurgery, Brigham and Women’s Hospital, Harvard Medical School, Boston, MA 02115, USA; jbernstock@partners.org; 4 Department of Anesthesiology and Intensive Care Medicine, University Hospital Bonn, 53127 Bonn, Germany; felix.lehmann@ukbonn.de; 5 Department of Neurology, University Medicine Rostock, 18055 Rostock, Germany; matthias.wittstock@med.uni-rostock.de; 6 Department of Neurology, University Hospital Bonn, 53127 Bonn, Germany; julian.zimmermann@ukbonn.de; 7 Department of Coagulation Disorders, University Hospital Frankfurt, 60590 Frankfurt, Germany; wolfgang.miesbach@kgu.de

**Keywords:** cerebral sinus and vein thrombosis (CVT), decompressive craniectomy, intracerebral hemorrhage (ICH), Covid-19, vaccination, SARS-CoV-2, vaccine-induced immune thrombotic thrombocytopenia (VITT)

## Abstract

Given the ongoing global SARS-CoV-2-vaccination efforts, clinical awareness needs to be raised regarding the possibility of an increased incidence of SARS-CoV-2-vaccine-related immune-mediated thrombocytopenia in patients with intracerebral hemorrhage (ICH) secondary to cerebral sinus and vein thrombosis (CVT) requiring (emergency) neurosurgical treatment in the context of vaccine-induced immune thrombotic thrombocytopenia (VITT). Only recently, an association of vaccinations and cerebral sinus and vein thrombosis has been described. In a number of cases, neurosurgical treatment is warranted for these patients and special considerations are warranted when addressing the perioperative coagulation. We, herein, describe the past management of patients with VITT and established a literature-guided algorithm for the treatment of patients when addressing the impaired coagulation in these patients. Increasing insights addressing the pathophysiology of SARS-CoV-2-vaccine-related immune-mediated thrombocytopenia guide physicians in developing an interdisciplinary algorithm taking into account the special considerations of this disease.

## 1. Introduction

Thrombosis of the cerebral veins and dural sinuses is a rare but serious event with an incidence of about 1.3 per 100,000 person-years [1], that can lead to a myriad of devastating effects within the central nervous system (CNS). Unfortunately, the pathogenesis of cerebral sinus and vein thrombosis (CVT) remains elusive. It is prudent to note that several clinical conditions have been associated with the development of CVT (e.g., intracranial surgery, tumor compression, and endocrine disturbances), Refs. [2,3,4] with CVT itself often resulting in intracerebral hemorrhage (ICH) [5].

After initial observation of only a few cases, increasing numbers of thromboembolic complications in patients receiving the SARS-CoV-2 vaccine have been published [6,7,8]. The pathogenetic mechanism underlying this condition termed vaccine-induced immune thrombotic thrombopenia (VITT) was identified by Greinacher and colleagues: The induction of anti-platelet-factor-4 (PF4) antibodies causing platelet activation and resembling a heparin-induced-thrombocytopenia-like disease [9,10]. This pathophysiologic mechanism implies potential relevant neurosurgical consequences that could be derived from at least the sequelae of CVT. The incidence of VITT is unknown and is described to affect only few patients among millions of vaccinated individuals [9] with the highest incidence described in Norway (1 in 26,000) [11]. With a condition only identified recently and only >200 cases identified, unfortunately only limited information on the outcome is available as of now. In many of those patients developing VITT, the cause of death was intracranial hemorrhage [12].

A number of medical approaches exist with regard to the clinical management of CVT, including intravenous heparin therapy [13], direct endovascular thrombolytic therapy [14], and/or the use of low molecular weight heparin (LMWH) [15]. In addition, patients who develop CVT with impending herniation may also be treated surgically via decompressive craniectomy (DC) [16]. When considering administration of anticoagulants or other supportive therapies designed to target systemic mediators of immune-mediated thrombocytopenia, the risks of intracranial hemorrhage vs. CVT progression must be weighed [17].

In this study, the authors shed light on the detrimental course that patients may take after admission for intracranial bleeding in the context of vaccine-induced immune thrombotic thrombocytopenia. Further, we propose specific actions to be taken and to be avoided by physicians of various specialties in the joint treatment of these complex patients.

## 2. Exemplary Case Presentation

**Figure 1:**
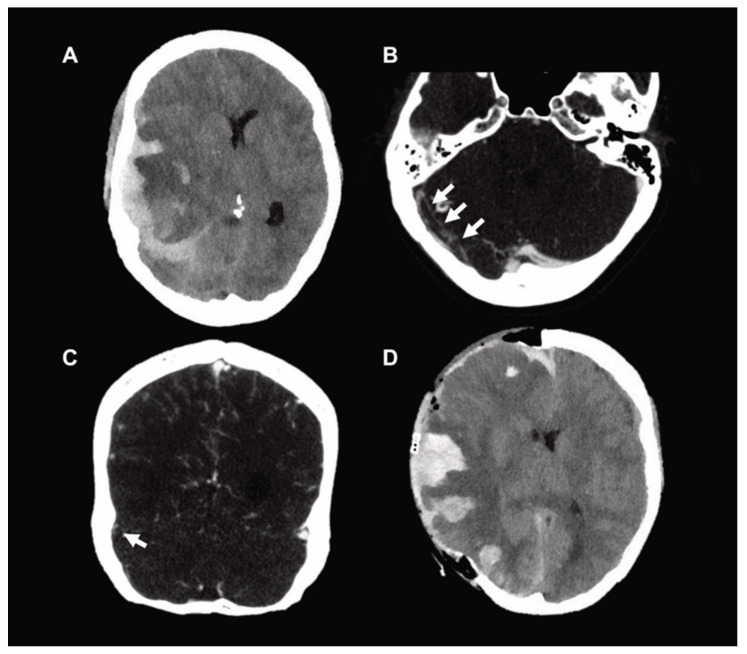
(**A**) Preoperative CT scan which demonstrates parenchymal stasis hemorrhage and a spontaneous acute subdural hematoma with associated midline shift and impending herniation. (**B**,**C**) Axial and coronal images, respectively, which demonstrate marked sinus vein thrombosis (arrows) on CT venography (CTV). (**D**) Postoperative CT scan after decompressive craniectomy.

Herein, the authors present a neurosurgical perspective on the management of three patients with CVT after vaccination with the ChAdOx1 nCoV-19 vaccine (AZD1222) and the Ad26.COV2.S vaccine (JNJ-78436735) for SARS-CoV-2. Ethical approval was obtained prior to data collection (IRB no. A2021 0112). All patients presented to the Emergency Department with progressive headaches at 7, 10, and 12 days, respectively, after the first vaccination; all three patients suffered rapid neurological deterioration within a few hours. Head computed tomography imaging including venograms revealed large-scale sinus thrombosis and ICH resulting in a mass effect with clinical signs of herniation warranting emergent surgical intervention (Figure 1). None of the patients reported here had a medical history of any known pre-existing disease and/or regular medication intake. In particular, there was no history of any pre-existing arterial and/or venous thrombotic disease/complications or hormonal therapy prior to the ictus. Further, no other venous thrombosis was diagnosed during the short period of hospitalization.

Laboratory values on admission demonstrated a substantial thrombocytopenia in all three patients (i.e., platelet count of 9 G/L; 24 G/L; 48 G/L respectively; Table 1).

Given that immune-mediated thrombocytopenia was suspected, therapy with intravenous immunoglobulin (IVIg at 1 g/kg) and corticosteroids was immediately initiated after consultation with the Hematology department as management was extrapolated from the treatment of immune HIT [18]. Given radiographic/clinical signs of impending herniation and the urgent need for decompressive surgery, platelets were given in all cases perioperatively despite the suspicion of anti-PF4 antibodies. Intraoperatively, severe bleeding and venous stasis still posed a challenge to the surgeon requiring immaculate hemostasis despite a cautious intraoperative strategy with an effort to avoid injury to the brain. In all three cases, the use of artificial hemostyptics and further transfusions controlled intraoperative bleeding, thereby allowing for sufficient surgical decompression of the affected hemisphere via DC. All patients were transferred to the Neuro-intensive care unit for further therapy postoperatively. Argatroban was employed for anticoagulation. In one patient, mechanical venous thrombectomy was performed by an interventional neuroradiologist in response to the postoperative increase in CVT noted on serial imaging. Postoperative/postinterventional imaging did demonstrate improvement of CVT but also highlighted the marked progression of ICH and resultant brain damage. Ultimately, all three patients succumbed to their extensive cranial injuries.

## 3. Discussion

**Figure 2:**
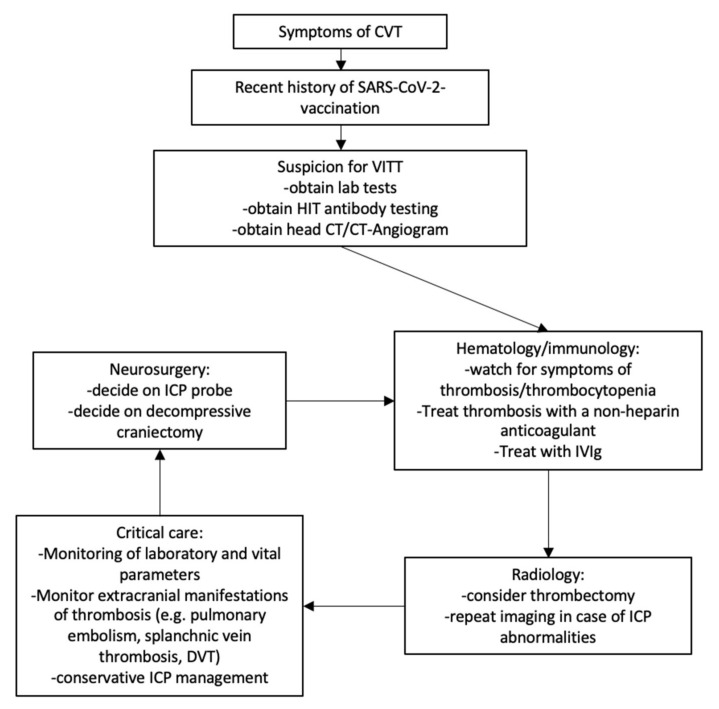
Proposed treatment algorithm in case of VITT-related CVT. When VITT is suspected in patients with symptoms of CVT, diagnostics verifying the diagnosis should be performed. While awaiting results of HIT antibody testing (PF4 ELISA and/or functional assay), patients should be empirically treated for VITT incorporating expertise from relevant specialties.

Surgical treatment of ICH related to venous stasis resulting from CVT remains challenging. To address such a challenging clinical situation, a rapid multidisciplinary approach that combines the expertise of a multitude of specialists including experts in hematology, immunology radiology, critical care, and neurosurgery is ultimately warranted (Figure 2).

### 3.1. Neurosurgical Considerations

The emergent presentation of a patient with the need for DC leads to the systematic execution of standardized neurosurgical procedures. Timely clarity about the coagulation situation is an important necessity for both the treating neurosurgeon and the physicians involved in the perioperative care of these patients. In the cases described above, thrombocytopenia was initially detected in the emergency routine laboratory. Due to the information given in the medical history about the recent SARS-CoV-2 vaccination, a corresponding association was suspected with regard to both CVT and thrombocytopenia. Nevertheless, the procedure of DC—especially in the presence of additionally elevated intravenous pressure (due to CVT)—represents a clear challenge to the perioperative management, also with regard to an optimal coagulation situation. While transfusion of platelets before surgery is critical for the neurosurgeon, the administration of platelets may actually be harmful in patients suffering from an immunological-related disease [19]. Recommendations regarding platelet transfusion in the context of VITT vary as general guidelines recommend that prophylactic platelet transfusions should be avoided but should be provided before interventions even in the case of suspected or diagnosed VITT [12]. However, platelet transfusions should only be executed after the administration of IVIg, if possible/permissible from a clinical point of view. Due to the clinical signs of herniation, a consideration of risks and merits was made, and platelet transfusion was performed to optimize intraoperative coagulation in the reported cases.

Further, secondary bleedings attributed to either progressive thrombosis and/or anticoagulation have been described in VITT and CVT [10]. The preoperative management of patients receiving anticoagulants may be of particular interest to the neurosurgeon. As described below, argatroban may serve as a suitable anticoagulant in these patients. The short half-life of argatroban (approximately 45 min) may allow for discontinuation of the anticoagulant as an adequate preoperative measure without the need for reversal of the anticoagulant function [20]. The postoperative management of anticoagulation in patients with ICH poses additional challenges to the physicians treating these patients. In a large study assessing the risk of patients with ICH and a high risk for thromboembolic events unrelated to VITT, the authors suggest an earliest starting point of therapeutic anticoagulation at day 6 [21]. In line with such observation, anticoagulation should be initiated postoperatively in CVT to prevent progressive thrombosis even in cases of ICH, the dosing, however, should be limited to preventive effects in the immediate postoperative course.

A unilateral hemicraniectomy, centered on the site of the largest hematoma and/or venous infarction may be considered as the goal of the surgery [22]. We believe that the recommendations for particular large hemicraniectomy (≥12 cm in diameter) for middle cerebral artery infarction/subarachnoid hemorrhage should also apply to CVT because lowering elevated ICP is the primary aim of DC [23]. Drug treatment of cerebral edema should be continued in the postoperative period and may also be guided by ICP monitoring. There are no definitive guidelines yet for ICP monitoring before or after DC. Postoperative imaging consists of head CT and CT-angiogram 24 h postoperatively or prior in case of perioperative complications and/or elevated ICP. The bone flap should be replaced once the brain swelling has subsided in order to avoid postoperative complications, which usually takes about three months [24,25].

### 3.2. Hematological Considerations

Previous observations have indicated that platelets serve an integral role in intercellular communication, mediating inflammatory and immunomodulatory activities beyond hemostasis and thrombosis [26]. Next to platelet activation, other lab findings such as thrombocytopenia, a reduction in fibrinogen, elevated D-Dimers, and circulating antibodies against platelet factor 4 (PF-4) may aid in diagnosing VITT [9,10,11,12]. In cases of thrombocytopenia and/or evidence of thrombosis, a test for heparin-induced thrombocytopenia (HIT-ELISA) should be performed, which is based on the immunological detection of antibodies against the complex of platelet factor 4 (PF4) and heparin. As a differential diagnosis of thrombocytopenia after vaccination, a secondary immune thrombocytopenia must be ruled out [7].

Current recommendations for VITT lab testing include the use of a sensitive, quantitative, immunologic test and avoiding rapid immunoassays [27]. A PF4/polyanion ELISA is the currently recommended screening test. If the PF4 test is positive, a classic HIPA test (HIPA, heparin-induced platelet activation) or a serotonin release assay (SRA, serotonin-release assay) may be additionally requested in the presence of proximate heparin exposure [9,11]. If a test is negative for PF4, VITT may still be considered as the underlying pathology for CVT depending on the patient history [28]. In line with this observation, the current definition of the CDC does not mandate positive PF4 findings in clinically conclusive cases.

Further laboratory diagnostics for VITT should always be carried out before IVIG is administered, as high doses of immunoglobulins can lead to a false negative test result. The following procedure should be followed in the event of a suspected VITT [29]: Rapid interdisciplinary coordination regarding further diagnostics (imaging), therapy and further inpatient care. Immediately stop any heparin therapy and start of immunomodulatory therapy with IVIG therapy 1 g/kg bodyweight for two days [29,30]. If thrombosis is present, initiation of anticoagulation may be warranted (after interdisciplinary consultation, depending on thrombocytopenia and imaging performed), mainly with argatroban (Argatra^®^). As an alternative to argatroban, bivalirudin, fondaparinux, and rivaroxoban were described as a potential treatment for VITT following the ChAdOx1 nCov-19 vaccination [9]. In particular, to further validate potential options to support impaired coagulation in patients with CVT/ICH and VITT (e.g., tranexamic acid, desmopressin, recombinant factor VIIa) [31], future studies addressing the potential systemic effects of SARS-CoV-2 vaccination on the coagulation system are clearly needed to help stratify risk and determine the optimal vaccination strategy for patient at risk for the development of VITT.

## 4. Conclusions

While the authors concur that any definitive association between vaccination and the development of CVT/ICH will require further study, we do feel such a relationship will be critical to exam in a blinded/prospective fashion. Given the ongoing global SARS-CoV-2-vaccination efforts, the present report is intended to raise clinical awareness regarding the possibility of an increased incidence of SARS-CoV-2-vaccine-related immune-mediated thrombocytopenia in patients with ICH/CVT requiring surgery, in an effort to optimize neurosurgical care.

## Figures and Tables

**Table 1:** Laboratory characteristics on admission. no. = number; NR = normal value range of the respective laboratory; yrs = years; PT = prothrombin time; INR = international normalized ratio; s = seconds; roTEG = rotational thromboelastography; R = reactive time; min = minutes; K = kinetic time; min = minutes; ME = maximum elasticity; h = hours; APRV = airway pressure release ventilation; fiO_2_ = fraction of inspired oxygen; pCO_2_ = partial pressure of carbon dioxide.

	Patient No. 1	Patient No. 2	Patient No. 3
**SARS-CoV2-vaccine**	ChAdOx1 nCoV-19	ChAdOx1 nCoV-19	Ad26.COV2.S
**age (yrs)**	47	50	44
**sex**	female	female	female
**medical history**	-	-	-
**medication prior to ictus**	-	-	-
**platelet count (G/L)**	9 (NR 150–370)	24 (NR 150–450)	48 (NR 150–370)
**time from vaccination to admission (days)**	12 days	7 days	10 days
**time from admission to first brain imaging (min)**	15 min	25 min	21 min
**PT**	10.7 s (NR 7.6–9.8)	-	8.6 s (NR 7.6–9.8)
**INR**	1.3	1.44	1.0
**activated partial thromboplastin time (aPTT)**	23.0 s (NR 25–35)	28 s (NR 27–37)	19.9 s (NR 25–35)
**thrombin clotting time**	20.9 s (NR < 20.5)	-	18.8 s (NR < 20.5)
**fibrinogen**	128 mg/dL (NR 180–355)	1.1 g/L (NR 1.8–3.5)	294 mg/dL (NR 180–355)
**roTEG R**	13 min (NR 8–16)	-	12 min (NR 8–16)
**roTEG K**	>60 min (NR 3–10)	-	10 min (NR 3–10)
**roTEG ME**	12 (NR 80–150)	-	52 (NR 80–150)
**D-dimer**	>35.2 mg/L (NR 0–0.5)	>35 mg/L (NR 0–0.5)	>35 mg/L (NR 0–0.5)
**preoperative ventilator settings**	APRV 18/10, 2.3 s/2.3 s, fiO_2_ 30%	BIPAP 17/7, fiO_2_ 50%	APRV 20/10, 2.5 s/2.5 s, fiO_2_ 30%
**preoperative pCO_2_ (mmHg)**	31.9 (NR 35–46)	34.8 (NR 35–46)	32.2 (NR 35–46)
**time from admission to death (h)**	39 h	49 h	20 h

## Data Availability

Data may be available upon reasonable request.
